# Multi-donor × elite-based populations reveal QTL for low-lodging wheat

**DOI:** 10.1007/s00122-022-04063-6

**Published:** 2022-03-21

**Authors:** M. Fernanda Dreccer, Bethany Macdonald, Claire A. Farnsworth, M. Valeria Paccapelo, Mary Anne Awasi, Anthony G. Condon, Kerrie Forrest, Ian Lee Long, C. Lynne McIntyre

**Affiliations:** 1 CSIRO Agriculture and Food, Queensland Bioscience Precinct, 306 Carmody Road, Saint Lucia, QLD 4067 Australia; 2 Department of Agriculture and Fisheries, Leslie Research Facility, Toowoomba, QLD 4350 Australia; 3 CSIRO Cooper Laboratory, University of Queensland Gatton Campus, Gatton, QLD 4343 Australia; 4 CSIRO Agriculture and Food, Building 101, Clunies Ross Street, Black Mountain, ACT 2600 Australia; 5 Agriculture Victoria Research, Department of Jobs, Precincts and Regions, Agribio, 5 Ring Rd., Bundoora, VIC 3083 Australia

## Abstract

**Key message:**

Low-lodging high-yielding wheat germplasm and SNP-tagged novel alleles for lodging were identified in a process that involved selecting donors through functional phenotyping for underlying traits with a designed phenotypic screen, and a crossing strategy involving multiple-donor × elite populations.

**Abstract:**

Lodging is a barrier to achieving high yield in wheat. As part of a study investigating the potential to breed low-lodging high-yielding wheat, populations were developed crossing four low-lodging high-yielding donors selected based on lodging related traits, with three cultivars. Lodging was evaluated in single rows in an early generation and subsequently in plots in 2 years with contrasting lodging environment. A large number of lines lodged less than their recurrent parents, and some were also higher yielding. Heritability for lodging was high, but the genetic correlation between contrasting environments was intermediate-low. Lodging genotypic rankings in single rows did not correlate well with plots. Populations from the highest lodging background were genotyped (90 K iSelect BeadChip array). Fourteen markers on nine chromosomes were associated with lodging, differing under high- versus low-lodging conditions. Of the fourteen markers, ten were found to co-locate with previously identified QTL for lodging-related traits or at homoeologous locations for previously identified lodging-related QTL, while the remaining four markers (in chromosomes 2D, 4D, 7B and 7D) appear to map to novel QTL for lodging. Lines with more favourable markers lodged less, suggesting value in these markers as a selection tool. This study demonstrates that the combination of donor functional phenotyping, screen design and crossing strategy can help identify novel alleles in germplasm without requiring extensive bi-parental populations.

**Supplementary Information:**

The online version contains supplementary material available at 10.1007/s00122-022-04063-6.

## Introduction

Lodging, the displacement of crop shoots from their vertical position, is a complex trait, both phenotypically (Dreccer et al. 2020) and genetically (Berry and Berry 2015). Over the decades, it has undermined breeders’ efforts to realise yield potential-related improvements (Foulkes et al. 2011). In wheat, lodging generally occurs after flowering and is detrimental to achieving high yields (Acreche and Slafer 2011), results in inefficient use of agricultural supplies such as fertilisers and water (Berry et al. 2004), and may trigger poor grain quality under certain environmental conditions (Fischer and Stapper 1987).

Lodging has been reported as a problem in crops other than wheat, such as rice (Ookawa et al. 2014), oilseed rape (Kendall et al. 2017; Wu and Ma 2016) and sorghum (Esechie 1983). Historically, in most crops, as widespread use of nitrogen fertiliser became available to increase production per unit area, a genetic control for height was sought to control the risk of lodging. This led to the adoption of the height-reducing genes during the first “Green revolution” (1966–1985), an era supported by large public investment in crop genetic improvement (Pingali 2012). In wheat, the use of gibberellin insensitive dwarfing genes *Rht-B1b* and *Rht-D1b* became widespread in breeding programs (Fischer and Stockman 1986), while in rice, the same was true for the semi-dwarf *sd1* mutation (Ookawa et al. 2010a). Carbon partitioned away from stems and into the harvested organs made higher yields possible, but at the same time, increased the leverage on the shoot and the plant. For this reason, high-yielding crops continue to be at high risk of lodging, and the challenge of breeding ever higher-yielding low-lodging crops persists, particularly in environments where the topsoil is wet due to rain or irrigation and/or wind speed is high after flowering and during grain filling. Plant growth regulators are an available agronomic intervention to manage lodging; however, unlike other inputs, appropriate choice of product and application timing is subject to substantial genotype by environment by management interactions (Peake et al. 2020) besides representing an additional cost. Another consideration is that further reduction in height may not be desirable. Aisawi et al. (2015) observed a trend towards higher biomass and yield in taller spring semi-dwarf cultivars in CIMMYT in a wide furrow cultivation system. Breeding lodging-tolerant varieties that are not necessarily short could represent a solution to further yield potential and maximise use of grain and straw without compromising standability. Straw is not only an important component of small-holders livestock systems globally, but also a commodity in its own right (Blümmel et al. 2020).

Phenotypically, lodging has been characterised as being underpinned by root-, stem- or shoot-related traits (Baker et al. 1998; Berry et al. 2003). Among the shoot-related traits, shoot leverage is conditioned by the height at the centre of gravity, the amplitude and frequency of movement in response to wind, the spike area and the wind gust speed (Baker et al. 1998). The role of supporting traits in terms of stem and anchorage attributes has been extensively quantified in rice and winter and spring wheat. In rice, stem characteristics such as material strength, based on lignin concentration and positioning and stem thickness, have been identified as pivotal to prevent lodging (Ookawa et al. 2010a, b). In wheat, stem strength plays a role, but root anchorage is also important (Berry et al. 2007; Dreccer et al. 2020; Piñera-Chavez et al. 2016). Root traits contributing to anchorage are the width and depth of the root plate spread. Both for stem and root traits, phenotyping is onerous in terms of time and labour and needs to be focused and optimised (Piñera-Chavez et al. 2020).

The unpredictable occurrence of lodging has made it difficult for breeders to select for lodging tolerance, and, ultimately, diagnostic genetic markers would be a useful tool to improve standability in a breeding program. Rice is the crop where the genetic regulation of traits conferring lodging tolerance has been studied in more detail and characteristics of the stem have emerged as most influential both for flooded and dry-seeded cultivation. QTL have been identified for culm diameter (Kashiwagi et al. 2008; Yadav et al. 2017), bending moment at breaking and section modulus (Ookawa et al. 2010a, 2016) and culm length and strength estimated with a push probe (Yadav et al. 2017), among others. QTL for similar traits have been found in wild rice (Long et al. 2020). In maize, stalks with higher flexibility are associated with resistance to lodging by wind and a QTL has been found for stalk fracture angle before tasselling (Wang et al. 2020) and others for brace-root development which help with anchorage (Sun et al. 2020).

In wheat, most DNA marker studies have been carried out in bi-parental populations. Berry and Berry (2015) studied the genetic basis of traits underpinning lodging in two winter wheat double-haploid populations with a composite marker, low-density map. The authors found that the main quantitative trait loci (QTL) effects for lodging resistance were for height, stem diameter, material strength and failure moment (13 QTL), and root plate spread and depth (4 QTL). Further work by Piñera-Chavez et al. (2021) in a subset of lines of the *Avalon x Cadenza* double haploid mapping population found 16 QTL, including nine for stem-related and two for root-related characteristics. In their study, 49.6% of phenotypic variation in stem strength was explained by QTL on chromosomes 1D and 3B, while 22% of phenotypic variation on root plate spread was explained by a QTL in chromosome 5B. Studying stem traits linked to lodging in a Chinese recombinant inbred line population, Song et al. (2021) identified 12 major QTL for eight traits in chromosomes 2D, 3A, 4B, 4D, 5A, 5B, 5D, and 6B, each QTL explaining from 0.6 to 34.6% of the phenotypic variation.

Within a broader objective to generate high-yielding low-lodging wheat, this study had two main aims. The first aim was to evaluate purpose-built populations for propensity to lodge, from the single row stage (BC1F4) to small plots in two subsequent years, under contrasting lodging pressure, using a method relying on fertilisation and overhead irrigation developed in a previous study (Dreccer et al. 2020). The populations resulted from crossing four low-lodging high-yielding donors selected for contrasting phenotype in lodging-related traits (Dreccer et al. 2020), with three Australian cultivars widely used in breeding. The purpose of this strategy was to evaluate the donor/s effect against the same and across backgrounds, not only because the expression of donor trait/genetic effects can differ in different backgrounds (Rebetzke et al. 2001, 2003), but also because results could be readily applied by industry. A second aim was to choose the populations from the background with highest propensity to lodge and identify genetic markers for lodging score under contrasting lodging pressure. More specifically, this study asked (1) if low-lodging high-yielding donors resulted in consistent performance across genetic backgrounds, (2) if lodging rankings were consistent in contrasting environments, (3) if lodging scores in single rows, as customary in early generation evaluations, were associated with lodging in plots, and (4) if QTL were consistent in environments with contrasting conditions leading to lodging.

## Materials and methods

### Populations: parents and donors

Twelve populations were derived from crosses between three adapted Australian cultivars as recurrent parents and four donors (Table 1). The donors were selected using data from 4 years of multi-environment evaluation in four locations in the Northern Cropping belt of Australia (Emerald: 23.59° S, 148.20° E; Gatton: 27.54° S, 152.34° E, Narrabri: 30.28° S, 149.80° E; Spring Ridge: 31.18° S, 150.42° E). Donors were high-yielding and low-lodging, combined with superior root plate spread and contrasting time to flowering, height and stem breaking strength when phenotyped for lodging and lodging-related traits in 2 years in a single location and analysed with a multi-trait and regression tree-type analysis, after extensive testing for lodging in multi-environment trials in the target population of environments (Dreccer et al. 2020) (Table 1). Parents and donors were genotyped for the presence of Rht-B1b and Rht-D1b (Ellis et al. 2002).

**Table 1:** Predicted mean of flowering in days after sowing, height, yield, lodging score, root plate spread and stem breaking strength and classification according to Fig. 5 based on trials in 2014 and 2015 in Dreccer et al. (2020), for recurrent parents (R. Parent) and donors of the populations

Genotype	Pedigree	Used as	Flowering (DAS)	Height (cm)	Yield (t ha^−1^)	Lodging score^a^	Root plate spread (mm)	Breaking strength (N)	Dreccer et al. (2020) Node # in regression tree analysis in Fig. 5, ^c^
Gregory	(DH)PELSART/3*BATAVIA	R.Parent	105 (1.9)	113 (1.9)	5.5 (0.4)	17.5 (4.6)	51 (2.8)	9.5 (1.0)	5.8^b^
Spitfire	DRYSDALE/KUKRI	R.Parent	99 (1.9)	97 (1.9)	5.9 (0.4)	4.6 (2.3)	53 (2.8)	8.6 (1.0)	5.7^b^
Suntop	SUNCO/2*PASTOR//SUN-436-E	R.Parent	96 (0.7)	104 (1.4)	6.1 (0.2)	2.7 (1.1)	54 (1.7)	10.7 (0.6)	2.5^b^
Cobra	WESTONIA/W-29	Donor	106 (0.7)	86 (1.4)	6.5 (0.2)	0.2 (0.3)	65 (1.7)	11.8 (0.6)	2.2
RTHiY32	WHEAR//2*PRL/2*PASTOR	Donor	102 (0.7)	105 (1.4)	6.5 (0.2)	3.6 (1.2)	63 (1.7)	10.0 (0.6)	2.2
RTHiY40	WBLL1*2/KUKUNA*2//WHEAR	Donor	95 (0.7)	114 (1.4)	6.8 (0.2)	1.1 (0.7)	66 (1.7)	14.1 (0.6)	2.2
RTHiY57	VAN'S'/3/CNDR'S'/ANA//CNDR'S'/MUS'S'/4/CHAM-6	Donor	96 (0.7)	111 (1.4)	6.3 (0.2)	1.1 (0.7)	62 (1.7)	9.1 (0.7)	2.2

Standard error of the mean between brackets. Each year had three replicates of each genotype

^a^Lodging score measurement description in (Dreccer et al. 2020) and Materials and Methods of this manuscript

^b^The line was classified in different nodes in the regression tree 2014 and 2015

^c^Regression trees were constructed to rank traits in their ability to predict lodging. The first number listed in the column corresponds to the node where the genotype clustered in a model including root plate spread, height at the centre of gravity, internode length and external diameter, wall thickness, stem breaking strength, spike area and spikes per plant. The second number corresponds to a node in a similar model where height to the centre of gravity was replaced by height to the ear tip and ear area was replaced by grain yield. Details about methods in Dreccer et al. (2020)

#### Populations evaluated in single rows

##### Trial design and management

To evaluate lodging in the early stages of population development, a single row evaluation trial was designed. A total of 2588 BC1F4 lines were sown on 25 July 2016 in single rows at the CSIRO Gatton Research Station, Gatton, QLD (27.54° S, 152.34° E, 89° masl). These lines were product from single seed descent, where extremely late or tall plants were discarded between F2 and F4. The paddock was fertilised with 300 kg N ha^−1^ pre-sowing to create conditions for lodging and irrigated intermittently to increase seed production. Plots were 1.5 m long, seven rows wide at 0.22 m spacing. To minimise the opportunity for lodging-susceptible lines to fall onto adjacent lines, the cultivar Cobra was sown in rows 1, 3, 5 and 7 within each plot as border, ensuring each test line had the same neighbours. Cobra is short thus minimising competition for light and has good standability (Table 1). Within the test rows, planting density varied from 17 to 23 pl m^−1^, equivalent to 75–105 pl m^−2^. Rows were hand-harvested.

The trial was designed as a partially replicated experiment latinized by ranges and runs (Cullis et al. 2006), with six incomplete blocks based on irrigation sections, in 105 ranges by 25 runs, each run with seven single rows of which three were test lines and four were border. Lines were randomly allocated to incomplete blocks in a way that ensured the number of lines in each of the 12 family groups (three recurrent parents × four donors) was balanced across the incomplete blocks. The design was generated using the design package Optimal Design (Butler 2009) in the R software environment (R Core Team 2021) to minimise prediction error variance for line comparisons.

##### Measurements

Single rows were observed for segregation of phenology and height, only uniform lines were assessed further, and 234 lines were discontinued. Crop development was assessed with a Zadoks score (Zadoks et al. 1974) on 6 and 19 October 2016; a score of 65 is equivalent to mid-flowering. Height was measured on more than ten replicates (single rows) per recurrent parent and donor. Based on these measurements, lines were classified into three categories: class 1 = shorter than the shortest parent, class 3 = taller than the tallest parent, 2 = between shortest and tallest parent. Lodging was scored as the angle of displacement from the vertical, where 0 is equivalent to plants standing (vertical).

##### Statistical analysis

The Zadoks scores were square-root-transformed to ensure homogeneity of variance and analysed using a linear mixed model. Lodging was analysed using a two-stage approach due to the high proportion of zeroes (i.e. not lodged). The first stage involved treating lodging as a binomial variable, where the response was lodged (1) or not lodged (0) using a generalised linear mixed model with a logit link. The second stage involved analysing only the data from rows that had a lodging score greater than zero in a linear mixed model.

In all models, lines were included as random effects. The line effects were partitioned into additive and non-additive line (genetic) effects, using the pedigree-based additive relationship matrix to account for relationships between lines (Oakey et al. 2006). Additive effects refer to the genetic component that can be attributed to the relationship between lines (breeding values) and non-additive refers to the remaining genetic component. A random term for the incomplete blocks was also included in the models. In the model for Zadoks score and the model in the second stage of the lodging analysis, the residual errors were modelled using a separable variance structure for ranges and rows, where each of the single rows within a plot was considered as separate rows, allowing the modelling of smooth spatial trends (Gilmour et al. 1997). Variance parameters were estimated using residual maximum likelihood (REML) estimation (Patterson and Thompson 1971). Prediction of additive and non-additive line effects was generated from the model as empirical best linear unbiased predictions (eBLUPs). Total genetic eBLUPs were calculated through the sum of additive and non-additive eBLUPs. The analysis was performed using ASReml-R (Butler 2021) in the R software environment (R Core Team 2021).

#### Populations evaluated in plots

##### Trial design and management

The twelve populations were sown at CSIRO Gatton Research Station as BC1F5 in 2017 and BC1F6 in 2018. Lines were grown in plots 4 m long, seven rows wide at 0.22 m spacing. The populations based on different recurrent parents were sown as advised for each recurrent parent by the National Variety Trials guide (Lush 2017) for the region (Gregory: 15-May-2017 and 13-May -2018, Suntop and Spitfire: 24-May-2017 and 23-May -2018). The site was fertilised to ensure the expression of high yield and lodging, with a total N target of 400 kg N ha^−1^ in 120 cm depth. The trials were irrigated after sowing to ensure establishment and intermittently before flowering and weekly after flowering with 25–40 mm to induce contrasting lodging depending on seasonal conditions. Weeds, pests and diseases were chemically controlled. Weather data were obtained from the local meteorological station (Australian Bureau of Meteorology, http://www.bom.gov.au/climate/data/, < 1 km from the site).

The trials were designed in three adjacent research paddocks, where each paddock was associated with a recurrent parent. This allowed adjacent paddocks to be sown at different times, enabling the populations to flower in the same time period according to their phenology. Each adjacent paddock was designed as a partially replicated experiment latinized by ranges and runs (Cullis et al. 2006) with two incomplete replicate blocks, where every line was grown at least once in the paddock of its recurrent parent. The replicated lines were randomly selected so that the number of lines replicated in each family were proportional to the overall family size. The seven parents and donors were each grown twice in every paddock, except in the Suntop paddock in 2018 where Cobra, Gregory, RTHiY40 and Spitfire were only grown once. A higher proportion of Gregory lines were discarded in the single-row selection process (2016) because they were too tall or too late, which resulted in unequal family size. As a result, more replication could occur within the Gregory paddock compared with the Suntop and Spitfire paddocks. The designs were generated using the design package Optimal Design (Butler 2009) in the R software environment (R Development Core Team 2016).

##### Measurements

Development was scored with the Zadoks decimal code (Zadoks et al. 1974) to determine flowering. Height to the tip of the spike was recorded shortly after flowering with three measurements per plot.

Each plot was assessed for percentage of the crop lodged, excluding border rows. This involves assessing the plot and dividing it in up to three homogenous sections, assigning the percentage of the total (100%) they each occupy and at which angle from the vertical the plants lean (Fischer and Stapper 1987). The lodging score is then calculated based on a weighted average:

1 $$\begin{aligned} {\text{Lodging }}\,{\text{score}} = & \left( {{\text{area}}1 \% \times \frac{{{\text{angle}} 1}}{90} } \right) \\ & + \left( {{\text{area}}2 \% \times \frac{{{\text{angle}} 2}}{90} } \right) \\ & + \left( {{\text{area}}3 \% \times \frac{{{\text{angle}} 3}}{90} } \right) \\ \end{aligned}$$

A lodging score equal to 0 meant that the crop did not lodge, while a score of 100 meant that the crop was completely lodged (flat on the ground). For example, if 80% of the crop was at 20° from the vertical, the score would be 17.8 if the remaining 20% was standing vertically and 37.8 if the remaining 20% was flat on the ground. Lodging scores were measured on a weekly to biweekly interval from flowering and were analysed as an average to better reflect the impact on yield loss during grain filling.

In both years, heavy rains occurred around harvest time. As a result, the trials could not be harvested in 2017; in 2018, lodging intensified with rains around maturity, and machine harvest went ahead after trimming one meter at each end of the plot, with some grain loss.

##### Statistical analysis

The experiments were designed so that some comparison between the adjacent paddocks would be possible; however, the data for the recurrent parents and donors showed large non-genotypic differences between the adjacent paddocks. Due to the limited number of lines present in common across paddocks, the underlying adjacent paddock difference could not be reliably separated from the paddock differences associated with the three recurrent parents.

The data collected in 2017 and 2018 were analysed together for each recurrent parent paddock. Flowering, height, lodging and yield (only 2018) were analysed separately for each paddock in a linear mixed model framework. Year was fitted as a fixed effect, while the interaction between line and year (genetic effect) was included as a random effect. The genetic (line) effects were partitioned into additive genetic effects and non-additive genetic effects, with the relationship between the lines taken into account through the additive relationship matrix based on the pedigree (Oakey et al. 2006). The additive and non-additive genetic variances were estimated separately for each year, with the additive and non-additive genetic covariances between years also estimated. These genetic covariances were tested using a log-likelihood ratio test, if not significant the trials were treated as independent for additive or non-additive effects (assuming covariance of zero). The additive and non-additive genetic correlations between years were calculated using the estimates of genetic variances and covariances. Experimental design terms were included separately for each year as random effects, and spatial variation was modelled following the procedure of Gilmour et al. (1997). The residual effects were modelled separately for each year using a separable variance structure, with a first-order autoregressive model in both the range and run directions. Diagnostic tools were used to assess spatial variation in the field, with formal tests used to determine whether terms accounting for this spatial variation should be included in the model.

Estimates of variance parameters were generated using REML estimation (Patterson and Thompson 1971). Best linear unbiased estimates (eBLUES) were obtained for fixed terms in the model, and eBLUPS were obtained for random effects using estimated variance parameters. Predictions of additive and non-additive performance for each line for both years were derived from their respective models as eBLUPs. Total genetic eBLUPs were calculated through the sum of additive and non-additive eBLUPs. Heritability was calculated following Cullis et al. (2006) as appropriate for partially replicated designs.

All analyses were performed using ASReml-R (Butler 2009) in the R software environment (R Core Team 2021). The agreement in ranking of genotypes between lodging scores in the 2017 and 2018 trials and probability of lodging and the leaning angle of the lines that lodged in the single row experiment was tested using the Spearman correlation.

#### Molecular data and QTL analysis

##### Molecular marker data

From the 12 populations available, only lines with a Gregory background crossed to the four donors (RTHiY40, RTHiY32, RTHiY57 and Cobra) were genotyped using the 90 K iSelect BeadChip (Wang et al. 2014) at Agriculture Victoria. This background was selected because of its high propensity to lodge. Data consisted of marker genotypes corresponding to 60,586 SNPs for 288 lines from the Gregory families. The number of lines genotyped in each family was proportional to its size, 36, 99, 72 and 81 when Cobra, RTHiY32, RTHiY40 and RTHiY57 were the donors, respectively. A quality control process was performed, removing markers with a missing rate above 20%. The remaining missing data were imputed using a k-nearest neighbours’ approach in the pedicure package (Butler 2019) in the R environment (R Core Team 2021). Any markers with a minor allele frequency lower than 5% were removed from the data. No heterozygote genotypes were found; however, during imputation some heterozygous genotypes were created. The final molecular marker data set consisted of genotypes for 288 lines and 13,867 SNPs. The final set of molecular markers was distributed across the 21 chromosomes according to their location in the International Wheat Genome Sequencing Consortium genome assembly of Chinese Spring v1.0 (IWGSC 2018) (Supplementary Fig. 1). Unfortunately, parental seed issues meant that we were unable to genotype the parental seed.

##### QTL analyses

The analyses to identify putative QTL for lodging score, height and flowering were performed for each trial separately using a two-stage approach. The first stage analysed the phenotypic data through a linear mixed model that included fixed line effects, random experimental design effects and spatial trends in the field, if present, following Gilmour et al. (1997). Variance parameters were estimated using REML estimation (Patterson and Thompson 1971). Line effects were generated from the model as empirical best linear unbiased estimators (eBLUEs). The predictions for each line generated from this model were then used in the second stage as the dependent variable, i.e. measurements of the trait of interest. The second stage involved performing the analysis to find associations between each trait of interest and the molecular marker data that allow for the identification of putative QTL.

Initially, the genome was scanned fitting a linear mixed model for every single marker using ASReml-R (Butler 2021). Apart from the fixed marker effect, the model included a fixed family effect to account for the known population structure. Gregory and the four donors were considered as additional families, resulting in nine families in total. A random polygenic effect was also included in the model, and the realized genomic relationship matrix (scaled cross-product of marker scores) was considered to account for the covariance between lines due to that polygenic effect. The association between the traits of interest and each marker was captured by the marker p value from each model or equivalently, the score calculated as the negative logarithm of the p value.

Once the most outstanding markers were identified (score > 3), the single marker model was extended to a multi-marker model that considered a fixed effect for every outstanding marker. To avoid collinearity issues in the multi-marker model, linkage disequilibrium (LD) between pairs of outstanding markers was measured by the *R*-squared statistic using the R package snpStats (Clayton 2019) and when *R*-squared > 0.9 only the marker with the highest score was included in the model. A backwards variable selection process was followed, and non-significant markers (alpha = 0.05) were removed from the model until only significant markers remained. These models were fitted using ASReml-R.

##### Comparative mapping of QTL

To compare QTL positions found in this study with QTL identified in recent publications, markers defining QTL were positioned on the IWGSC v1 genome assembly where possible. Some publications note the wheat genome sequence position of QTL markers. For those markers without IWGSC v1 positioning, we used GrainGenes (wheat.pw.usda.gov/GG3), Pretzel (https://plantinformatics.io) (Keeble-Gagnère et al. 2019) and DAWN (Watson-Haigh et al. 2018) to identify the mapped position of a DNA marker or to obtain the sequence and blast the sequence against the IWGSC v1 wheat genome assembly. Pretzel was used for comparative map construction to investigate both homologous and homoeologous map locations for markers from different maps and chromosomes.

## Results

### Populations evaluated in single rows

**Fig. 1:**
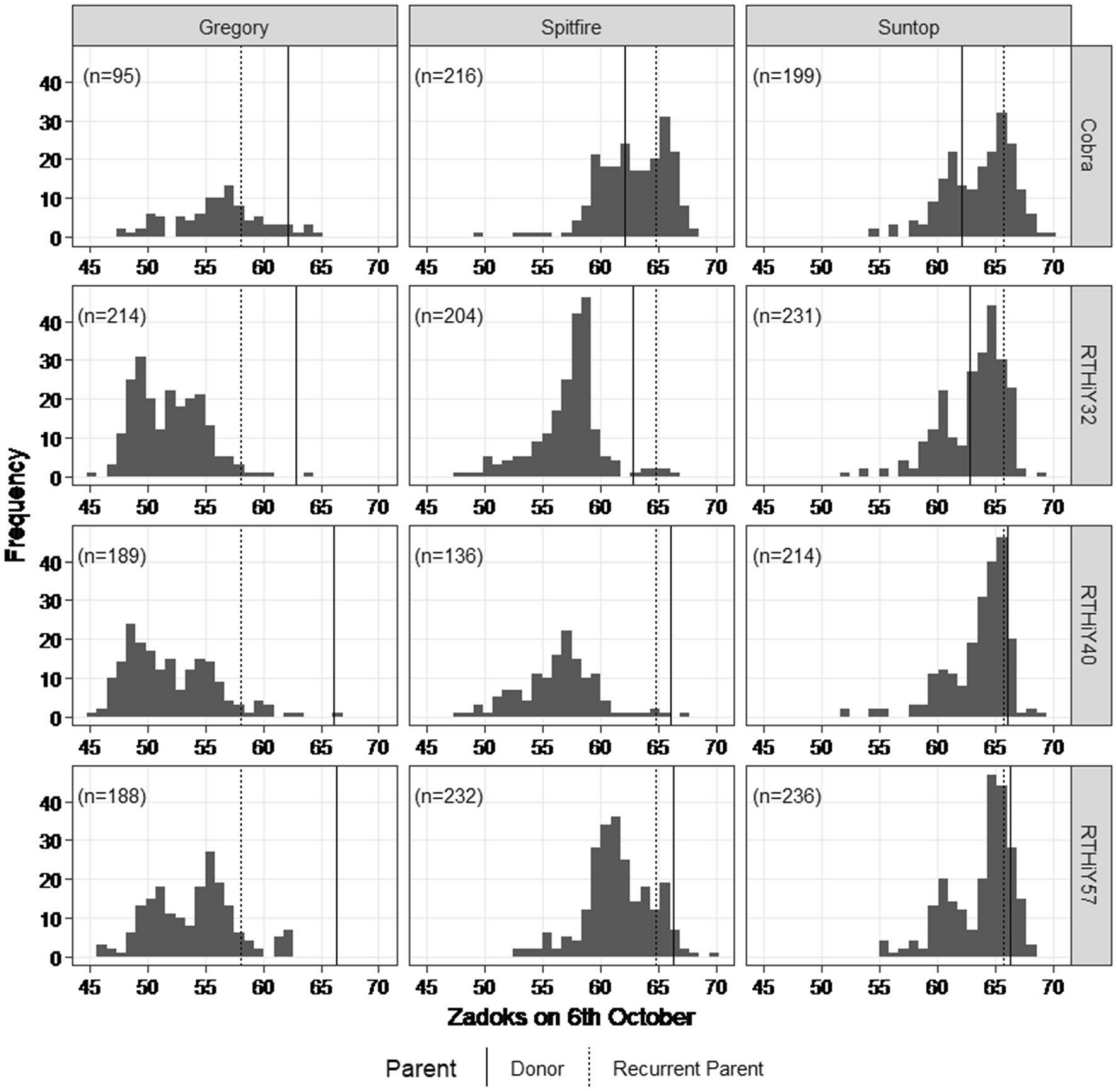
Distribution of Zadoks score for single rows on 6 October 2016. Vertical panels are the recurrent parents, horizontal panels are the donors, and n is the number of lines in each population. A lower Zadoks score is indicative of later flowering

The twelve BC1F4 populations were evaluated in single bordered rows in 2016 for development stage (Zadoks score), height category and angle of displacement from the vertical. When scored on 6 October (74 days after planting), many of the BC lines were developmentally less advanced than the recurrent parents or donors, particularly in the Gregory-derived populations and some of the Spitfire-derived populations, expressing transgressive segregation (Fig. 1).

Recurrent parents and donors varied for the presence of the major dwarfing genes *Rht-B1b* and *Rht-D1b*. Recurrent parents Gregory and Suntop and donors RTHiY32, RTHiY40 and RTHiY57 were homozygous for *Rht-B1b*, while recurrent parent Spitfire and donor Cobra were homozygous for *Rht-D1b.* There was substantial height variation in crosses segregating for these genes. About 92% of the BC lines fell within the intermediate-height Class 2 category (Table 2). Only a small proportion of BC lines fell into the taller than the tallest parent, Class 3. Gregory crosses to Cobra and to RTHiY40 had the highest proportion of tall lines, whereas Spitfire*2/RTHiY32, Spitfire*2/RTHiY40 and Suntop*2/Cobra had ca. 10% or more lines shorter than Cobra, the shortest parent (Class 1), some potentially double-dwarfs (*Rht-B1b* + *Rht-D1b*).

**Table 2:** Percentage of lines per height category by population in 2016 single bordered rows trial

Lines	Class 1 (%)	Class 2 (%)	Class 3 (%)	Number of lines	Height^a^ (cm)	*Rht* genotype
Gregory	0.0	93.9	6.1	114	114.3	*Rht-B1b*
Gregory*2/Cobra	2.4	75.6	22.0	82		
Gregory*2/RTHiY32	0.0	94.2	5.9	188		
Gregory*2/RTHiY40	1.3	82.5	16.2	154		
Gregory*2/RTHiY57	1.3	92.1	6.6	152		
Spitfire	0.7	98.6	0.7	144	97	*Rht-D1b*
Spitfire*2/Cobra	0.9	98.1	0.9	212		
Spitfire*2/RTHiY32	9.7	89.2	1.1	185		
Spitfire*2/RTHiY40	10.1	87.2	2.8	109		
Spitfire*2/RTHiY57	0.0	96.3	3.7	217		
Suntop	0.0	100.0	0.0	90	101	*Rht-B1b*
Suntop*2/Cobra	16.8	79.5	3.7	161		
Suntop*2/RTHiY32	0.5	94.8	4.8	210		
Suntop*2/RTHiY40	1.0	92.0	7.0	201		
Suntop*2/RTHiY57	0.0	91.4	8.6	220		
Cobra	0.0	100.0	0.0	120	83.6	*Rht-D1b*
RTHiY32	0.8	99.2	0.0	124	108.5	*Rht-B1b*
RTHiY40	0.0	88.2	11.8	119	111.5	*Rht-B1b*
RTHiY57	0.0	92.4	7.6	119	118.6	*Rht-B1b*

Class 1 = shorter than Cobra (83.6 cm); Class 3 = taller than RTHiY57 (118.6 cm); Class 2 = between Cobra and RTHiY57, height and *Rht* genotype of donors and recurrent parents

^a^Average of ten rows across the field measured to establish classes for height

**Fig. 2:**
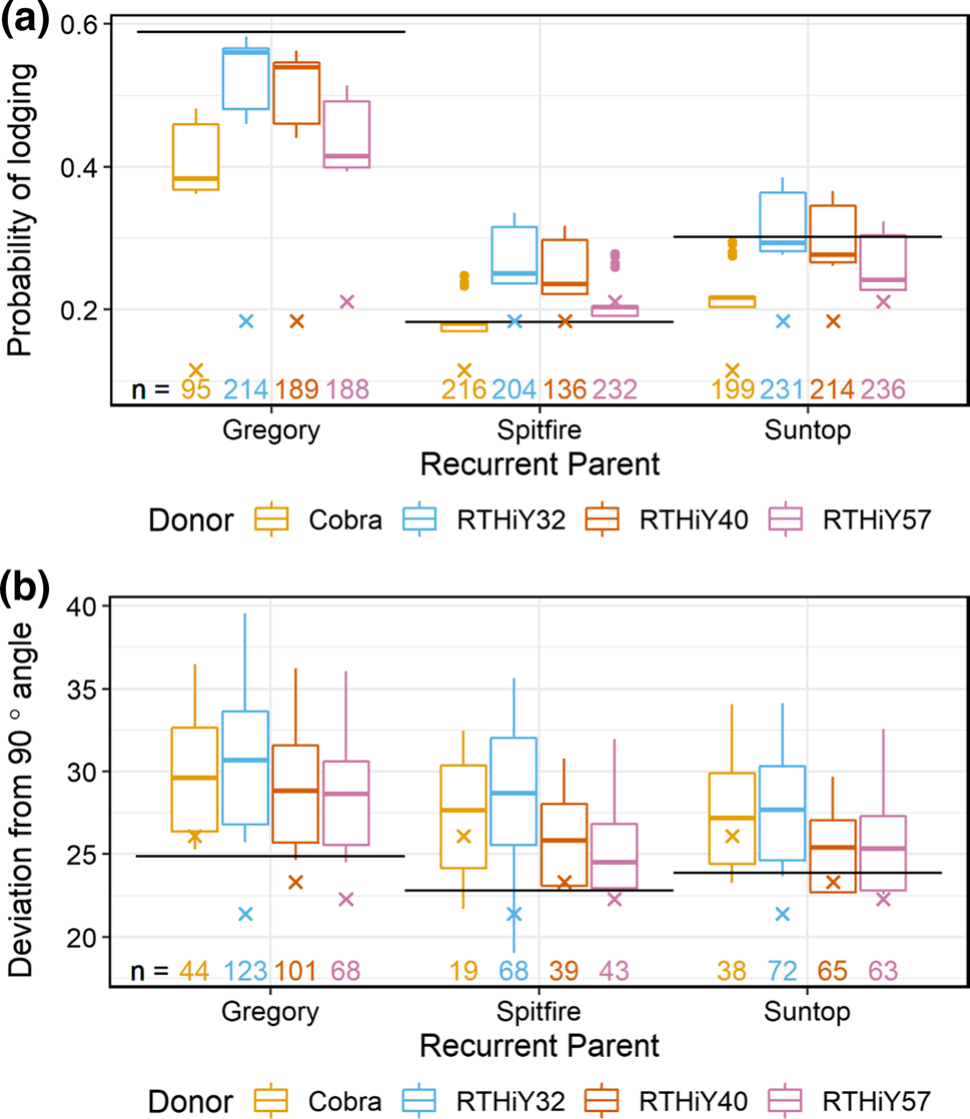
Probability of lodging (**a**) and lodging angle (deviation from vertical) (**b**) for BC lines that showed lodging when grown in 2016 single-bordered rows. Values are total genetic effects centred around the experiment mean. Black lines indicate trait values for the recurrent parents, and crosses indicate trait values for the donors

As BC lines were sown as bordered single rows and not as full plots, scoring of lodging was done based on the angle of displacement from the vertical. Due to the high proportion of zeros (no lodging), this lodging score was analysed as the probability and angle of lodging (see Materials and Methods). Among the recurrent parents, the probability of lodging was highest in Gregory, followed by Suntop and Spitfire (Fig. 2a). Cobra, the shortest donor, was the most effective in reducing lodging probability across recurrent parents, followed by RTHiY57, the tallest donor. Approximately 50% of the Gregory-derived BC lines showed some lodging, whereas for Spitfire and Suntop-derived lines this proportion was considerably lower, ca. 20–35%. For the proportion of lines that did show some lodging, median angle of lodging was slightly higher for the Gregory derivatives, followed by Spitfire and Suntop lines (Fig. 2b).

### Populations evaluated in field plots

#### Weather and irrigations

**Fig. 3:**
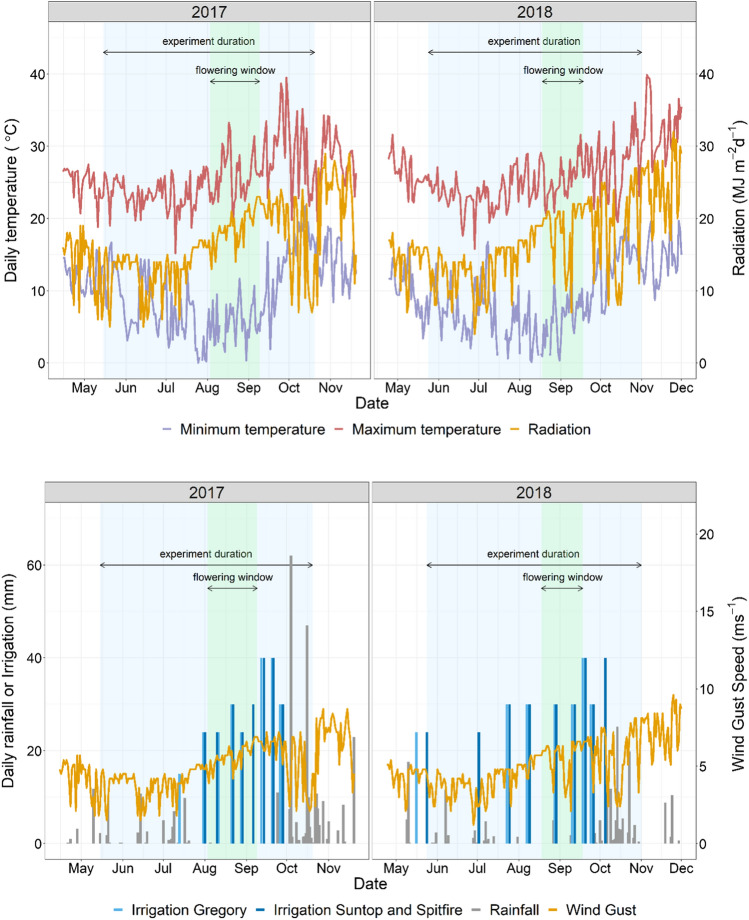
Minimum and maximum temperature and global radiation (top panels) and rainfall, irrigation and wind gust speed at 2 m height, (bottom panels). Irrigation indicated separately for Gregory as sown earlier; 30 mm irrigation on 20/09/2018 was only delivered to Suntop, not Spitfire

The objective of managing the irrigation was to achieve a high yield potential but also elicit lodging, and therefore irrigation was applied just before flowering and more frequently during grain filling. Between rainfall and irrigation, the Gregory blocks received a total of 479 mm in 2017 and 359 mm in 2018 (Fig. 3). The Spitfire and Suntop blocks received 452 mm of combined rainfall and irrigation in 2017, but in 2018, Spitfire received 393 and Suntop 423 mm. The irrigation was managed to create contrasting degrees of lodging conditions for the crops in each year, i.e. if in first year there was a large amount of lodging, less water was applied in the second year and vice versa. Wind gust speed at 2 m height increased during the season with a similar profile in 2017 and 2018.

#### Lodging

**Fig. 4:**
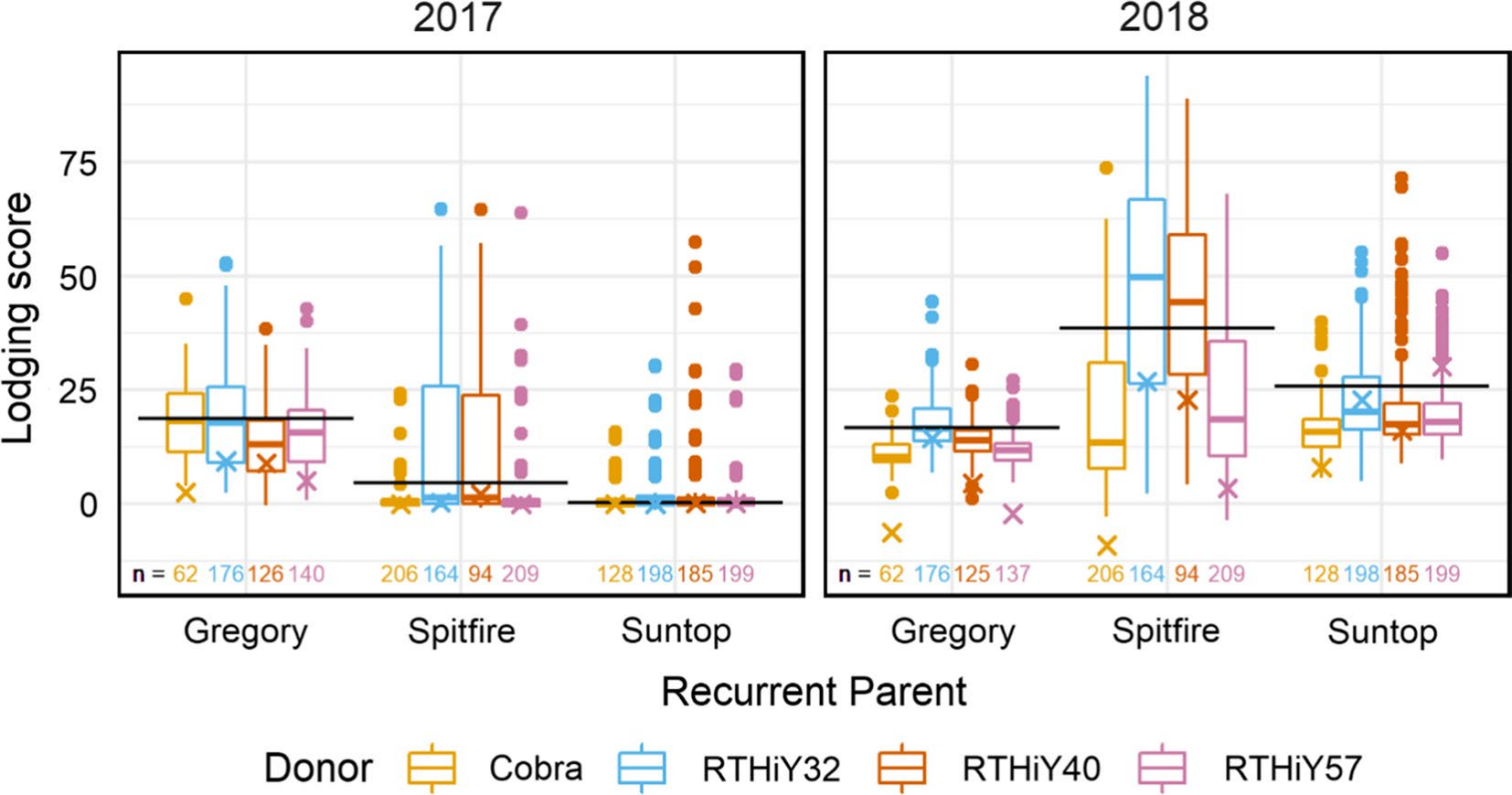
Lodging score in Gregory, Spitfire and Suntop populations. Values are the total genetic effects centred around population means. Horizontal lines are recurrent parent effects; crosses are donor effects. Number of lines evaluated per group at the bottom of graph

Heritability for lodging varied between 0.76 and 0.82 in the Gregory background, 0.84 and 0.97 in Spitfire, and 0.80 and 0.88 in Suntop (Table 3). Total genetic correlations between years were 0.5 and 0.6 in Gregory and Suntop and lower in Spitfire (0.32). The additive genetic correlation between years was high for Gregory, intermediate for Suntop and not estimated for Spitfire populations for which years were treated as independent as the additive covariance between years was not significant (Table 3). The contrasting lodging between years is the result of managing irrigation differently to create contrasting degrees of lodging, e.g. Gregory populations had high lodging in 2017 and were irrigated less in 2018 (Figs. 3 and 4).

**Table 3:** Number of lines evaluated in 2017/2018, mean, standard error (SE), heritability (*H*^2^) additive and total genetic correlations between years for lodging scores, flowering in days after sowing (DAS), and height (cm) in 2017 and 2018

Trait	Populations with recurrent parent	Line number^c^	2017			2018			Genetic correlation	
			Mean	SE	*H*^2^	Mean	SE	*H*^2^	Additive	Total
Lodging score	Gregory	504/500	16.3	1.5	0.82	14.5	0.7	0.76	0.88	0.60
	Spitfire	673/673	5.8	0.5	0.97	30.8	0.9	0.84	^b^	0.32
	Suntop	710/710	2.2	0.5	0.88	20.6	0.9	0.80	0.37	0.51
Flowering (DAS)	Gregory	501/500	104.5	0.2	0.92	104.8	0.4	0.91	0.99	0.94
	Spitfire	672/673	92.0	0.1	0.93	100.9	0.6	0.89	0.92	0.87
	Suntop	710/710	88.6	0.3	0.88	98.0	0.2	0.88	^b^	0.96
Height (cm)	Gregory	504/500	99.3	0.6	0.85	102.0	1.0	0.79	0.94	0.79
	Spitfire	673/673	86.3	0.5	0.92	91.3	0.5	0.93	0.94	0.89
	Suntop	708/708	98.5	0.5	0.86	98.4^a^	–	–		

^a^No genetic variance. Data average provided

^b^Additive covariance between years not significant. Additive effects between years were treated as independent (assuming covariance of zero)

^c^“/” separates years

Overall, 132 and 34 Gregory-derived lines lodged significantly less than Gregory (*p* < 0.05, data not shown) in 2017 and 2018, respectively, with the tallest donor, RTHiY40, producing phenotypes as effective as those derived from the shortest donor Cobra in both years. When Spitfire was the recurrent parent, 301 and 257 lodged less than Spitfire in 2017 and 2018, respectively (*p* < 0.05, data not shown); in particular, those derived from crosses to Cobra and RTHiY57 (contrasting in height), were best at reducing lodging (Fig. 4). Suntop populations hardly lodged in 2017, but 127 lodged less than the recurrent parent in 2018 (*p* < 0.05, data not shown) in response to increased irrigation. The proportion of additive genetic variance was higher in 2018 (95, 73 and 48% in Gregory, Spitfire and Suntop, respectively) versus 2017 (48, 57 and 14% in Gregory, Spitfire and Suntop, respectively) (Supplementary Fig. 2a).

#### Flowering

**Fig. 5:**
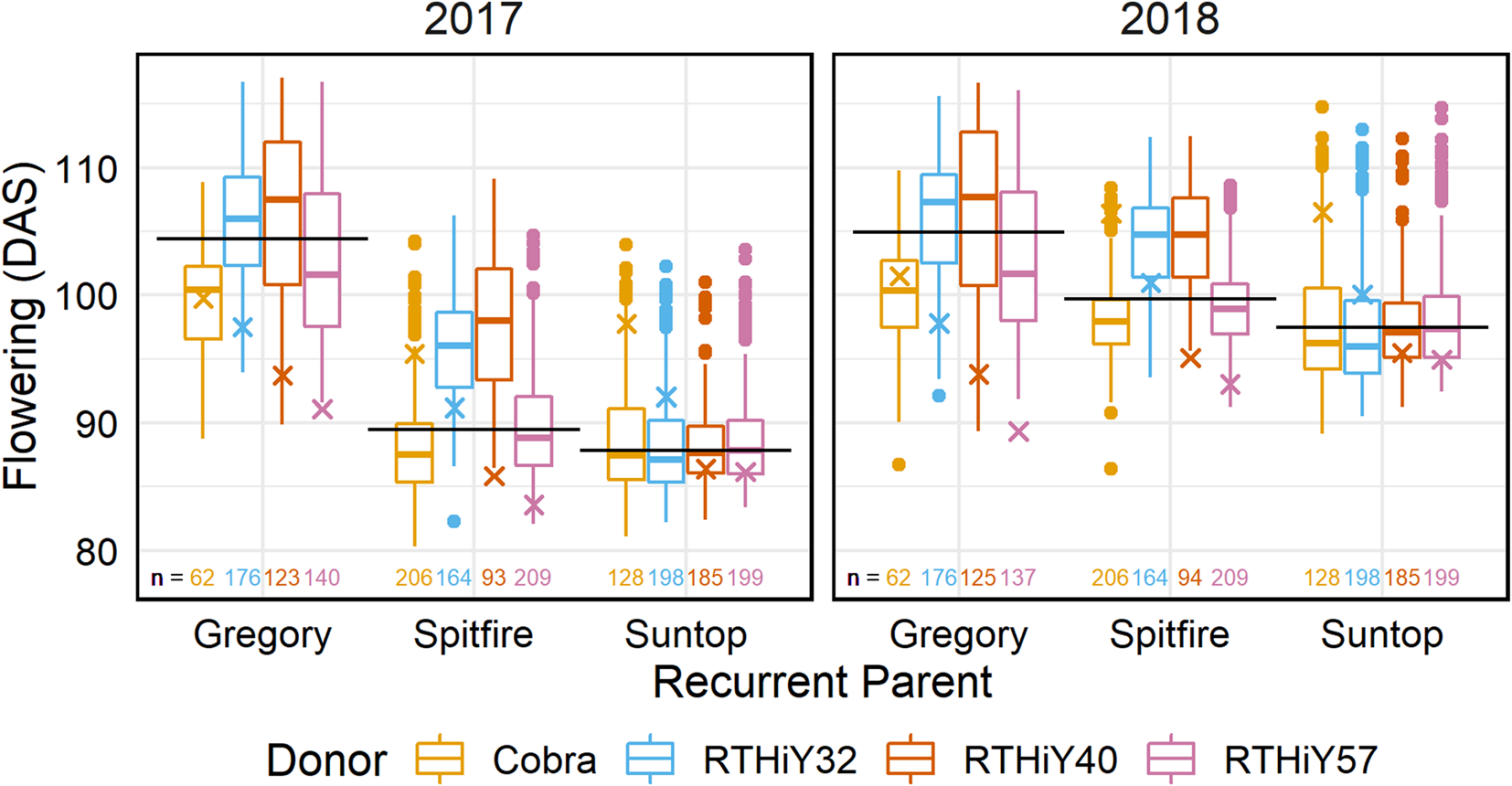
Flowering time in days after sowing (DAS) for Gregory, Spitfire and Suntop populations. Values are the total genetic effects centred around population means. Horizontal lines are recurrent parent effects; crosses are donor effects. Number of lines evaluated per population at the bottom of graph

Flowering had high heritability among the populations of each recurrent parent background (Table 3). Total genetic correlations between years were between 0.87 and 0.96, while additive genetic correlations were above 0.90 for Gregory and Spitfire populations but not estimated for Suntop as years were treated as independent (Table 3). Gregory-derived populations tended to flower ca. 15 days later on average than those derived from Suntop and some of the Spitfire-derived populations (Fig. 5). Amongst the Gregory and Spitfire derivatives, the populations with Cobra and RTHiY57 as donors had the largest proportion of lines that flowered earlier than the recurrent parents. In turn, Gregory or Spitfire crossed to RTHiY32 and RTHiY40 as donors had a large proportion of lines that flowered later than the recurrent parent. The median of the Suntop-derived populations coincided with the score for Suntop. Similar trends were observed in the additive component of the total genetic variation (Supplementary Fig. 2b).

#### Height

**Fig. 6:**
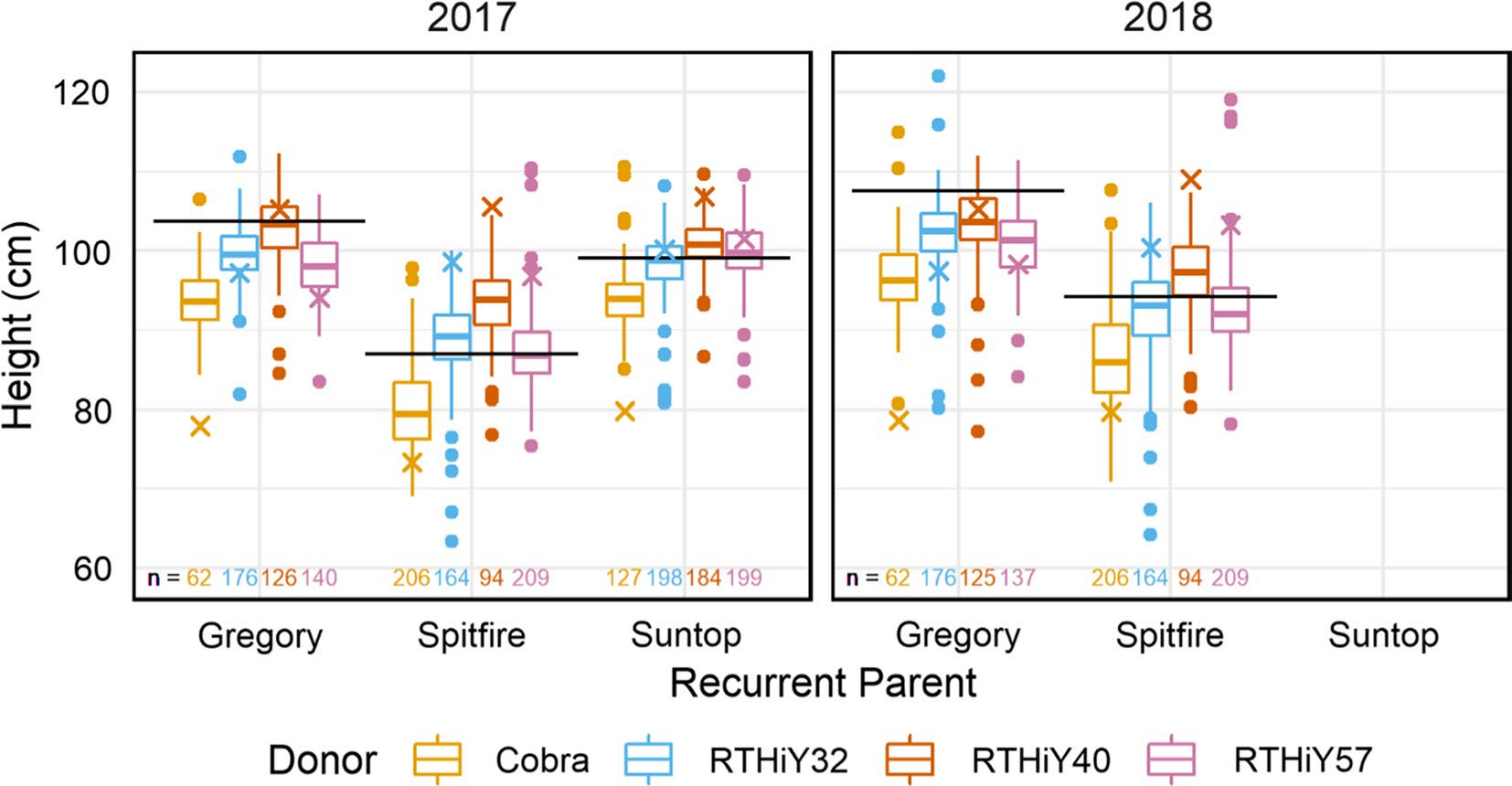
Plant height (cm) for Gregory, Spitfire and Suntop (2017) populations. Suntop-derived populations did not show genetic variation for height in 2018 (population mean = 98.4 cm). Values are the total genetic effects centred around population means. Horizontal lines are recurrent parent effects; crosses are donor effects. Number of lines evaluated per group at the bottom of graph

Height had high heritability among populations of each recurrent parent background. For Suntop in 2018, heritability could not be estimated due to lack of genotypic variance (Table 3). Total genetic correlations between years were ca. 0.80 or above, while additive genetic correlations were above 0.90 for Gregory and Spitfire (Table 3). Cobra was the best donor in terms of effective height reduction (Fig. 6) and at passing on the short height (additive effects) (Supplementary Fig. 2c). Between 75 and 100% of lines with Cobra as donor were shorter than the recurrent parent, on average by ca. 10 cm in Gregory, 6–7 cm in Spitfire and 0–5 cm in Suntop backgrounds. At the opposite end, crosses to RTHiY40, the tallest donor (106.4 cm), resulted in the highest proportion of lines taller than the recurrent parents in 2017 and in the Spitfire background in 2018. RTHiY57 (98.8 cm) was second to Cobra in effectiveness to reduce the height of BC lines when Gregory and Spitfire were recurrent parents (Fig. 6).

#### Yield in 2018

**Fig. 7:**
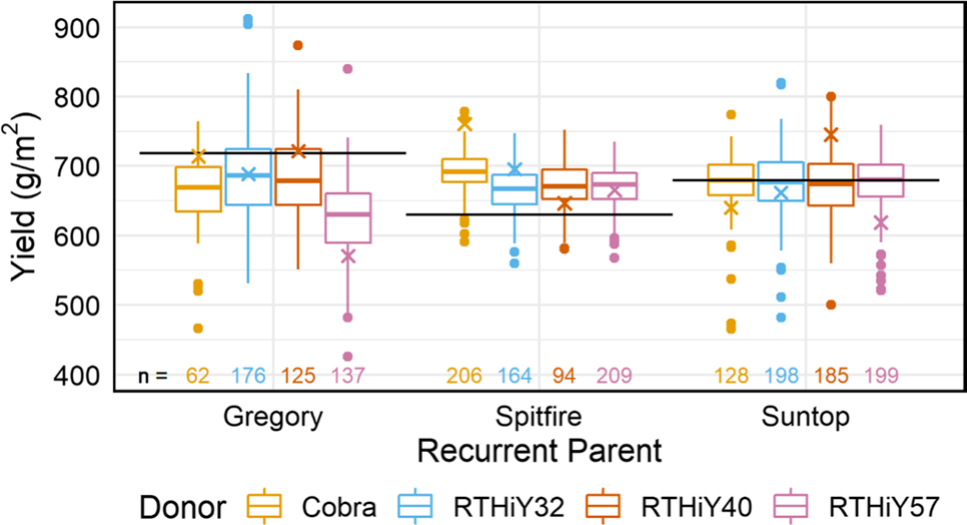
Machine yield (moisture corrected) in Gregory, Spitfire and Suntop populations in 2018. Values are the total genetic effects centred around population means. Horizontal lines are recurrent parent effects; crosses are donor effects. Number of lines evaluated per group at the bottom of graph

The yield for the different families ranged is presented in Fig. 7. The broad sense heritability for yield was 0.63 for Gregory, 0.43 for Spitfire and 0.55 for Suntop. Many lines would be better parents than the recurrent parents amongst the Gregory populations based on additive genetic effects (Supplementary Fig. 2d). Twelve Gregory-derived lines that lodged significantly less than the recurrent parent also yielded significantly more, four from the Cobra and eight from the RTHiY57 crosses (*p* < 0.05, data not shown). Similarly, four lines from the Suntop × Cobra cross that lodged significantly less than Suntop yielded significantly more (*P* < 0.05, data not shown).

### Association between single rows and plots and between different traits measured in plots

The relationship between lodging scores in the 2017 and 2018 plot trials, and the probability of lodging and the leaning angle of the lines that lodged in the single row experiment was estimated using the Spearman correlation. The overall correlation, between 2017 and 2018 lodging scores in plots and single rows, was 0.25 for probability of lodging and 0.19 for leaning angle. When each year and family was considered separately, the correlation between the probability of lodging in single rows and lodging scores in plots was 0.35, 0.36 and 018 for Gregory-, Spitfire- and Suntop-derived populations, respectively, in 2017, and 0.49,0.52 and 0.33 for Gregory-, Spitfire- and Suntop-derived populations, respectively, in 2018. The correlation between the leaning angle of the lines that lodged in 2016 bordered single rows and lodging scores in plot evaluation was 0.15, 0.38 and 0.15 for Gregory-, Spitfire- and Suntop-derived populations, respectively, in 2017 and 0.33, 0.42 and 0.11 for Gregory-, Spitfire- and Suntop-derived populations, respectively, in 2018.

**Fig. 8:**
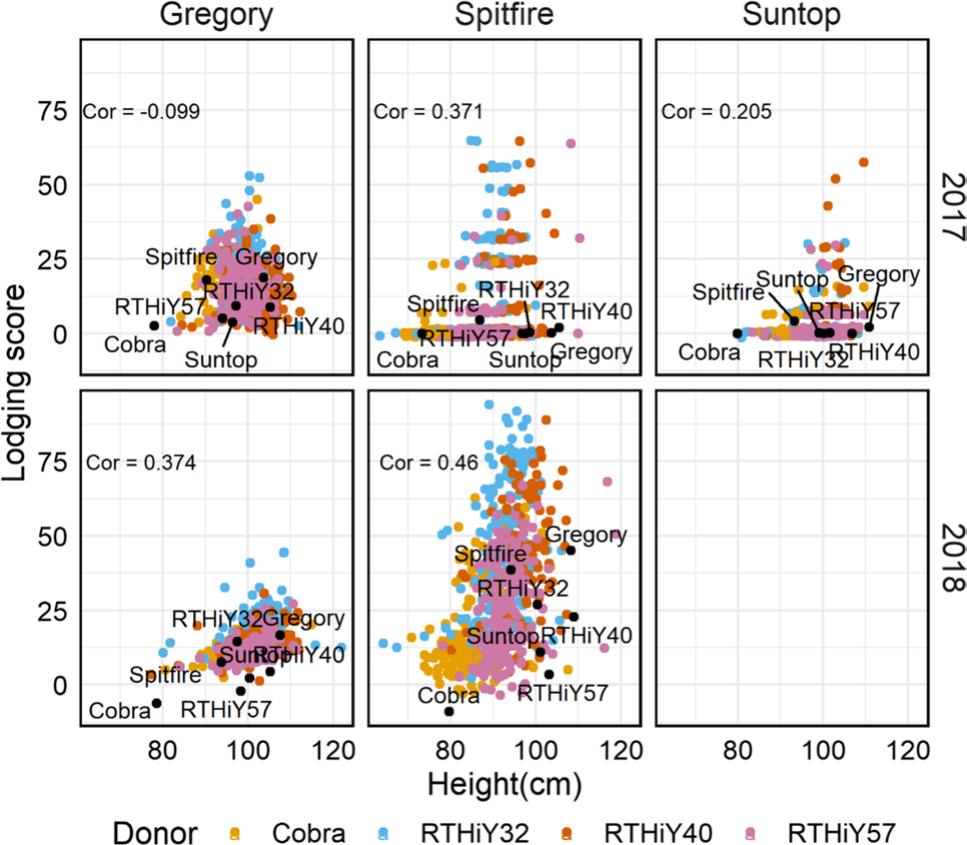
Lodging score against height in 2017 and 2018 for populations with recurrent parents Gregory, Spitfire and Suntop. For each trait, values are the total genetic effects centred around population means. There was no genetic variation in Suntop populations in 2018

In the current study, there was no evidence of a direct relationship between lodging and height in either year despite the variation in height introduced with the choice of donors (Fig. 8). There was also no evidence of a relationship between lodging and phenology in general or lodging and yield in 2018 (Supplementary Fig. 3).

### QTL Identification

For Gregory populations, the association between lodging score and SNPs along the whole genome in the initial single marker scan is represented by Manhattan plots shown in Supplementary Fig. 4(a) 2017 and (b) 2018. The number of selected putative QTL found to be significant at a level of alpha = 0.05 for lodging score, once the linkage disequilibrium criteria and backward selection were applied, was six in 2017 and eight in 2018, years of high and low lodging, respectively.

In 2017, three QTL were detected on chromosome 7D and one each in chromosomes 4B, 5B and 6B (Table 4). Four QTL had positive effects and two negative effects. Markers with a positive effect indicate that lodging would increase by this amount when both alleles are the reference SNP allele (1; AA) and decrease by this amount when both alleles are not the reference SNP allele (-1; BB). The opposite is true for markers with a negative effect. A QTL on 7D explained ca. 10.0% of the genetic variance and one on 5B explained ca. 9.2%. These QTL showed an estimated allele effect of 4.64 and 4.54 on lodging score, respectively, increasing lodging. From the four significant QTL that explained 4.4% or less of the genetic variance, two decreased lodging in 7D and 6B, and two increased it in 4B and 7D, with absolute allelic effects ranging between 2.5 and 3.1 in lodging score.

**Table 4:** Selected putative QTL for lodging score in 2017 and 2018 showing the SNP name, chromosome (Chr) position in IWGSC genome assembly of Chinese Spring v1.0, SNP effect size and its standard error (SE), significance (*p* value) and per cent of genetic variance the marker accounts for (%vaf)

Year	SNP name	Chr	Position	Effect	SE	*p* value	%vaf
2017	BobWhite_c8415_728	7D	13,610,617	4.64	0.1886	0.0106	10.0
	wsnp_Ex_c47152_52446529	5B	476,636,741	4.54	0.2098	0.0148	9.2
	D_contig55669_249	7D	630,707,433	− 3.10	0.1333	0.0144	4.4
	BobWhite_c15529_288	4B	66058159^a^	3.06	0.1189	0.0090	4.3
	Ex_c3405_203	6B	903614^b^	− 2.77	0.0925	0.0046	3.6
	BS00011583_51	7D	22,528,719	2.50	0.0985	0.0117	2.9
2018	wsnp_CAP11_c1196_692246	7B	451,078,548	*− 0.2138*	*0.1647*	0.0021	30.2
	Excalibur_rep_c102984_157	2D	641,109,645	*− 0.1482*	*0.0931*	0.0002	14.2
	Tdurum_contig22253_104	3A	495024538^c^	*0.1455*	*0.0915*	0.0002	13.9
	wsnp_Ex_c12450_19850827	4D	124260727^d^	*0.1321*	*0.1041*	0.0026	11.1
	RAC875_rep_c77148_311	3A	56,399,941	*0.1025*	*0.0874*	0.0052	6.9
	BS00071655_51	6A	23,437,723	*− 0.0974*	*0.0969*	0.0164	5.9
	Tdurum_contig9934_103	7B	112,890,988	*0.0902*	*0.0962*	0.0249	5.6
	Excalibur_c30739_72	^e^		− *0.0909*	*0.0755*	0.0042	5.5

Note that in 2018 the lodging score was transformed (logit, in italics) and effects and SE are reported in logit scale. QTL are sorted by %vaf

^a^Inferred position based on LD. This SNP has been mapped to 4B in several bi-parental populations (Wang et al. 2014)

^b^Inferred position based on LD. This SNP mapped has been mapped to 6D (pos. 3,976,771) in the IWGSC Chinese Spring reference genome version1.0 (IWGSC 2018)

^c^Shows homology to other locations, genetically mapped only to 3A

^d^Inferred position based on LD. This SNP has been genetically mapped in bi-parental populations (Wang et al. 2014) and LD to 6D (inferred position 470,684,296)

^e^Maps to multiple locations

In 2018, from the eight QTL, known positions were detected at chromosome 7B (2), 2D, 4D and 3A (2) and 6A, and one of unknown position (Table 4). Four QTL had positive effects and four negative effects. A putative QTL in 7B had the largest effect decreasing lodging and explained 30.2% of the genetic variance with high LOD. Two other QTL explained 14.2% and 13.9% of the genetic variance, one in 2D decreasing lodging and the other in 3A increasing lodging, respectively.

Comparative mapping of the markers in Table 4 suggests that the majority of markers are detecting separate loci, with two exceptions. The two markers that map to 6B (2017) and 6A (2018) are located at syntenic regions of these two chromosomes and may be associated with the same homoeologous locus. In 2017, markers BobWhite_c8415_728 and BS00011583_51 in map within 9Mbp on chromosome 7D and may be associated with the same locus; marker D_contig55669_249 also maps to 7D but to a different region of 7D. The two 7B markers map to different regions of 7B and neither region appears syntenic with the mapped markers on chromosome 7D.

**Fig. 9:**
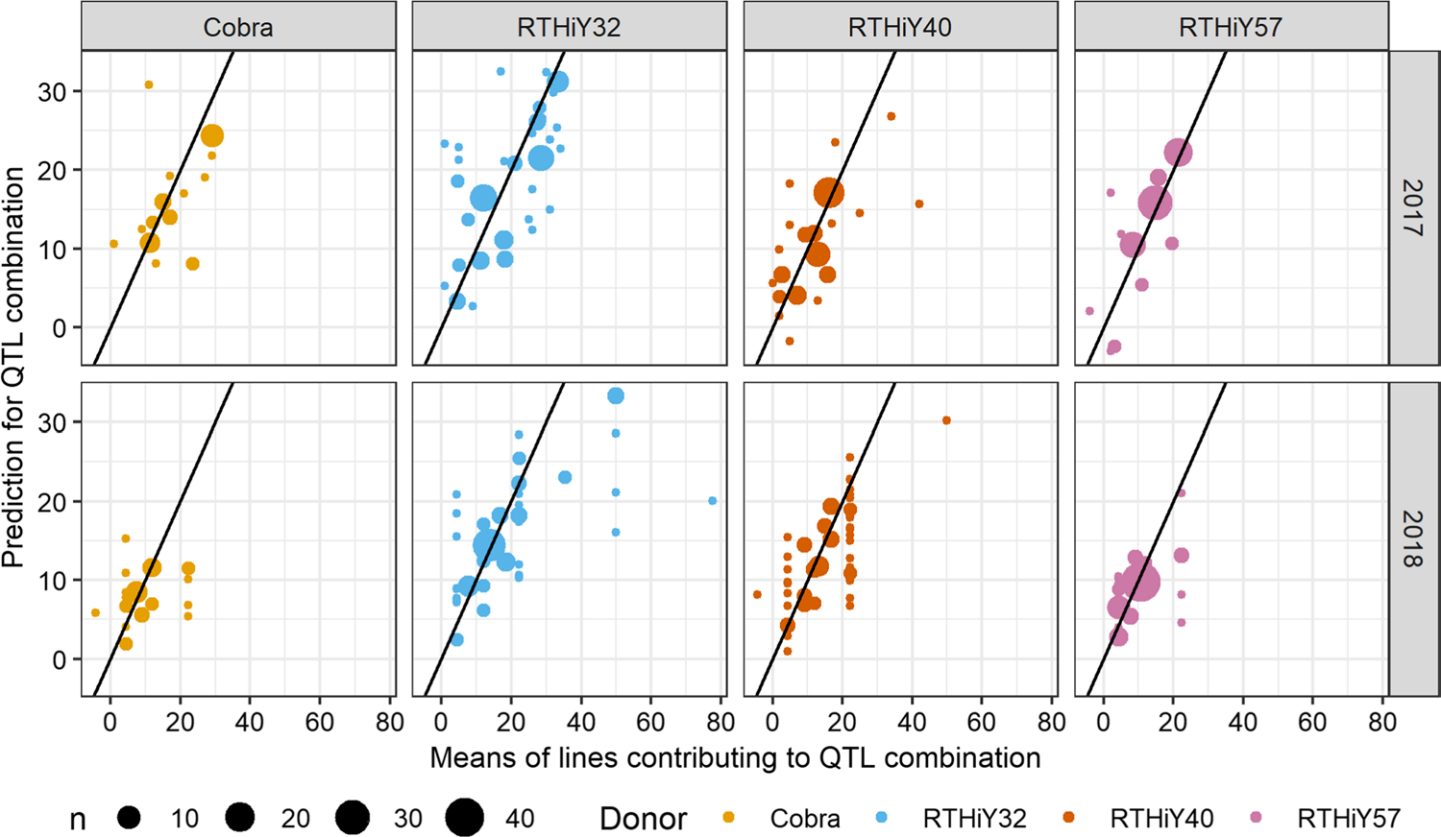
Predicted lodging score using family and putative QTL effects from the final model against observed lodging score means for each QTL combination in 2017 and 2018

For every family, all possible QTL profiles, i.e. all the genotype combinations for the putative QTLs, were considered for further investigation. When a QTL profile was represented by at least one line in the family, the mean of the observed phenotypic values was compared to the predicted lodging score based on the family effect and putative QTL genotypes (Fig. 9). Some QTL profiles were represented by more lines than others; hence, a weighted Pearson correlation (Emad and Bailey 2017) was considered as a measure of association between the predicted and observed values within families. In 2017, the weighted correlation values were 0.69 (Cobra), 0.71 (RTHiY32), 0.65 (RTHiY40), and 0.86 (RTHiY57); however, some QTL profiles were poorly represented in the families and the predicted value does not represent the means well. For 2018, predictions were back-transformed to the lodging score scale for interpretation purposes in Fig. 9. This year of trials showed a reasonable level of association between the predicted values and means but lower than 2017 (weighted correlation values on the logit scale were Cobra = 0.34, RTHiY32 = 0.72, RTHiY40 = 0.66 and RTHiY57 = 0.62). Since eight putative QTLs were included in 2018, the number of lines for every possible QTL profile decreased in comparison with 2017 where six QTLs were detected. The predicted values for all combinations of the putative QTL per year are included in Supplementary Tables 1 (2017) and 2 (2018) and the linkage disequilibrium between the putative QTL in 2017 and 2018 in Supplementary Table 3.

Putative QTL detected for height and flowering were investigated (Supplementary Table 4). Putative QTL detected for flowering in Chromosome 5A with LOD > 10 were very close to *VRN-A1* and *TaPhyC*, involved in the sensing of cold and long days, respectively, and known for their roles in regulating flowering time. Linkage disequilibrium was calculated between each lodging putative QTL and all the height and flowering QTL. Marker Tdurum_contig22253_104 identified on 3A for lodging score in 2018 was linked to two putative QTL on 3A (Excalibur_c3657_745 and Tdurum_contig10572_580) both with a small effect on plant height.

**Fig. 10:**
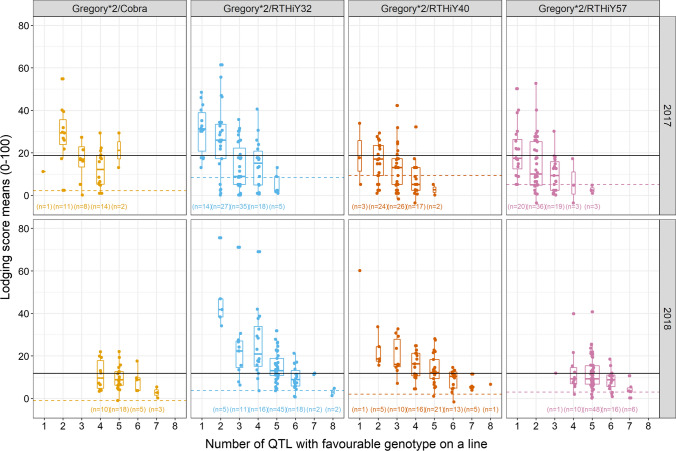
Lodging score raw means versus number of QTL with favourable genotype present in a line organised by family and year. Recurrent parent lodging score mean is represented with a black horizontal line, while donor means are represented by dashed lines. *n* is the number of lines that have a given number of QTL with favourable genotype

Finally, lodging scores are illustrated as a function of the number of favourable alleles present by family (Fig. 10). In general, the lines with lower lodging score have an increasingly greater number of favourable alleles.

## Discussion

This study demonstrates that the combination of donor functional phenotyping, screening design and crossing strategy can lead to identifying novel alleles without requiring the development of extensive bi-parental populations. The approach was effective and economical, as there are less lines for trait phenotyping at the diversity panel evaluation stage.

### Donor choice, screen design and crossing strategy helped identify high-yielding, low-lodging germplasm

Breeding high-yielding, low-lodging wheat is a complex breeding objective, not only because lodging is underpinned by different plant characteristics and influenced by the environment, but also because higher yields increase the chance of lodging. A previous study identified four donors with good standability and high yield, high root plate spread and contrasting height (Dreccer et al. 2020) that were crossed in this study to three commercial varieties, to allow for comparisons of donor effects across genetic backgrounds. Evaluation was carried out with the management criteria developed during the selection of donors, i.e. sown in a representative location to the target population of environments with heavy clay soils, under a high-fertility regime and irrigation, as described in Dreccer et al. (2020). Additionally, the populations with the same recurrent parent were sown at the optimal sowing date for the recurrent parent, allowing for independent management of irrigation in relation to crop stage and weather. Unfortunately, sowing the populations in adjacent paddocks limited the chance to compare donors across recurrent parent backgrounds more directly despite repeated check lines sown across, when we could not separate the underlying paddock differences from the paddock differences associated with the recurrent parents. However, the screening method proved effective in allowing contrasting conditions to be created in a minimum time, e.g. for the Gregory derived populations the high lodging in the first year was balanced with observations of lower lodging the following year by irrigation management to test the hypotheses about lodging ranking and QTL consistency across environments. This type of screening is an intermediate step between relying on weather in multiple locations (e.g. National Variety Trials networks) and having maximum control over the stress imposed at a particular scale, e.g. blasting water with a large fan (Shrestha et al. 2020). It is reliable and easily achievable in a farm with access to irrigation (Dreccer et al. 2020) and the trial itself could be amenable to high-throughput phenotyping with an unmanned aerial vehicle (Singh et al. 2019b; Zhao et al. 2020). Others have proposed high-density planting under irrigation to select against lodging in winter wheat (Xiao et al. 2015).

Evaluation of the 12 BC populations under conditions contrasting for lodging showed many lines that lodged less than the respective recurrent parents, from those, some yielded more, but no selection pressure for yield was applied. In terms of selecting lines to go forward in a program when the recurrent parent has high-lodging scores, comparison against a stringent fixed threshold is worth considering, e.g. a lodging score of 17.8 (80% of the crop at 20° from the vertical, and the remaining 20% standing vertically). When we applied this criterion to our populations, we found that the number of lines with lodging score significantly lower than 17.8 was 146, 571 and 691 in Gregory, Spitfire and Suntop backgrounds, respectively, in 2017, and 100, 13 and 7 in Gregory, Spitfire and Suntop backgrounds in 2018. Ideally, both approaches should be compared before a final selection of lines is made. The heritability of lodging score was high in each trial × background combination, consistent with previous studies (Dreccer et al. 2020; Singh et al. 2019a).

Donors were mostly consistent in terms of performance across backgrounds, even when they cannot be directly compared. Families derived from Cobra, the shortest donor, and RTHiY57, the second tallest, were highly effective at reducing height across backgrounds and reduced lodging in Spitfire across lodging intensities. However, when Gregory was the recurrent parent, RTHiY40, the donor producing the tallest family, was the most effective at reducing lodging in a high-lodging trial (2017), while Cobra and RTHiY57 were most effective in a low-lodging year (2018). Overall, height range and rankings were similar across environments, but lodging was not. This led to a lack of association between lodging and height in these populations, suggesting that other traits may be contributing to standability, particularly under high lodging pressure, and opening the possibility to develop wheat crops that produce higher yield and straw biomass, suitable for dual purpose. The genetic correlation for lodging scores between environments was intermediate in populations in Gregory and Suntop background and low in Spitfire and is also evidence of a complex interaction with the environment. Others have reported significant genotype by environment interactions for lodging in a range of wheat cultivars and populations (Piñera-Chavez et al. 2016, 2021).

### Single rows versus plots

Evaluating progeny attributes in early generations is an ideal opportunity to narrow down the number of lines for more extensive and costly evaluation, i.e. increasing genetic gain per unit cost. Fischer and Rebetzke (2018) mention three issues that hamper early generation selection as (a) lack of enough seed, (b) allele segregation and recombination before homozygosity is reached and (c) genotype by environment interactions conducive to low repeatability. In relation to evaluating a complex trait such as grain yield or related traits in early generations, they point to evidence where the phenotype of plants grown in isolation is not related to performance in a canopy. Others have shown how genotypes can differ in yield-related attributes in response to spacing (Sukumaran et al. 2015). In the case of lodging, it is an accepted practice amongst plant breeders to discard lines that “lean” on others in early generations, when they are grown in single rows. To prevent a line falling over and pushing an unrelated line, the single row evaluation in this study was designed so each line was bordered by the same short variety and the trial was statistically designed to obtain means that could be compared with plot results. Interestingly, genotypic rankings in lodging evaluated in single rows bordered by the same cultivar did not correlate well with those emerging from evaluation in plots. This highlights that when breeders discard lines that bend or lodge in early generations, they may not be effectively selecting the best performing under canopy conditions. Since discarding material in later generations due to lodging is a rather costly exercise for a breeding program, early selection for a trait/traits underpinning lodging and monitoring molecular markers represent an option, leaving the phenotyping for underpinning traits to the stage of diversity identification to select donors for the crossing program (Dreccer et al. 2020).

### QTL associated with lodging tolerance differ under high- and low-lodging conditions

This study identified 14 markers associated with lodging in 2 years of field trials contrasting in lodging pressure. These markers were distributed on nine chromosomes and were largely independent from flowering time and height. All 14 markers were mapped to the wheat genome using the IWGSCv1 reference genome to enable comparison with QTL identified in recently published papers. Of the 14 markers, ten were found to co-locate with previously identified QTL for lodging-related traits or at homoeologous locations for previously identified lodging-related QTL, while the remaining four markers appear to map to novel QTL for lodging. Some markers also appear to map close to some agronomic traits.

The largest two QTL identified in the high lodging 2017 trial were on Chromosome 7D and 5B and map to the same location as QTL for root plate spread in Berry and Berry (2015) and Piñera-Chavez et al. (2021). The marker on 7D (BobWhite_c8415_728) maps about 22Mbp from *Wx-D1*, a locus encoding granule-bound starch synthase which is a key enzyme in amylose synthesis. The marker on 5B (wsnp_Ex_c47152_52446529) maps in the vicinity (approximately 17Mbp away) of *Fr-B2 (FROST RESISTANCE 2),* a gene which affects frost tolerance, days to heading and yield (Eagles et al. 2016; Pearce et al. 2013). *VRN-B1* (*VERNALIZATION 1*) (Tóth et al. 2003), which has been shown to pleiotropically influence root attributes in wheat and barley (Arifuzzaman et al. 2014, 2016; Voss-Fels et al. 2018), is a further 8Mbp distal to our marker. Interestingly, Zhu et al. (2014) reported significant interactions between *VRN-A1* and *FR-A2*. None of these markers were associated with flowering.

Some QTL on Group 3, 6 and 7 chromosomes in the present paper appear to co-locate with or be homoeologous to QTL identified in other papers. For example, in the present paper, RAC875_rep_c77148_311 (2018) maps to a region on chromosome 3A close to the genome sequence position of a QTL for stem diameter identified by Berry and Berry (2015) and to a QTL cited by Song et al. (2021, Table S1). This marker maps close to Excalibur_c30739_72, from the low-lodging year (2018), both positioned between *Mft-A1*, associated with seed dormancy and pre-harvest sprouting, and *TaGIGANTEA* (*TaGI*), the latter known to influence development (Zikhali et al. 2016). The two loci detected on 6A and 6B in the present study appear to map to regions similar to or homoeologous to regions identified by Atkinson et al. (2015) and Piñera-Chavez et al. (2021) on chromosomes 6B and 6D related to rooting attributes, while the two regions identified by Berry and Berry (2015) on chromosomes 6A and 6B may also be homoeologous. The marker on 6A in our study (BS00071655_51) maps near to a major seed protein locus, *Gli-*6A (Halstead-Nussloch et al. 2021). There appears to be two major homoeologous loci clusters for lodging in Group 7 chromosomes. Three markers from this study and QTL detected in the paper by Berry and Berry (2015) map two different homoeologous regions on chromosomes 7B and 7D. Of particular interest is that two starch biosynthesis genes, *SSII* and Wx, map close to these regions. The marker Tdurum-contig9934-103 on 7B (2018) maps near *SSIIa-B1*, a starch synthase gene which contributes to the elongation of amylose chains in non-storage tissues and has been shown to be expressed in culms (Vrinten and Nakamura 2000); its role in stem strength or root-related traits is not known. Similarly, BobWhite_c8415_728 and BS00011583_51 map to a different region on chromosome 7D, not far from the location of *Wx-D1*, which encodes the amylose synthesis gene *GBSS,* and *VRN-D3*. In addition, in the present study, marker BobWhite_c15529_288 mapped to chromosome 4B. However, this marker also maps to chromosome 7B, near *VRN-B3*. This region may be homoeologous to the lodging region on 7D in the region of *VRN-D3*.

Four markers detected in the present study, including the 7B marker that explained the largest proportion of genetic variance detected in the lower lodging trial in 2018 (wsnp_CAP11_c1196_692246), appear to detect novel QTL for lodging. Two of these four markers map to chromosomes 7B and 7D to different locations to the two loci clusters referred to above. The third 7D marker detected in the 2017 trial (D_contig55669_249) maps to a new and unique location on chromosome 7D, while the 7B marker detected in the lower lodging trial in 2018 (wsnp_CAP11_c1196_692246) maps to a new location on chromosome 7B. The 4D marker identified in a low-lodging year trial maps to a region that has not been linked to lodging before and does not map near *VRN-D2* on 4D. However, this marker has been mapped previously to chromosome 6D and, in that study, it was associated with flag leaf attributes (Wu et al. 2016). Finally, the QTL on 2D (Excalibur_rep_c102984_157) has not been previously associated with lodging. However, in this study, the marker also mapped to chromosome 2A and was significantly associated with height. On 2D, the marker does not map near any known height genes, including Rht8 (Ellis et al. 2005). These regions could be further targeted for increasing lodging resistance.

QTL in a year with high lodging were different to those associated with standability in a low-lodging year, suggesting that the combination of traits that are important to promote standability under contrasting lodging pressure may be different. The difficulty at validating predictions between years supports this hypothesis and the need to carry out independent analyses per year/environment when conditions for lodging are contrasting. Both the studies by Berry and Berry (2015) and Piñera-Chavez et al. (2021) determined QTL based on mean data across the different environments despite significant GxE in the traits measured. In contrast, studying stem traits linked to lodging in a Chinese recombinant inbred line population, Song et al. (2021) identified 12 major QTL for eight traits, with only five present across different environments, in chromosomes 2D and 4D. Since lodging is a complex trait and can be quantitatively described, it is possible that different traits, and hence QTL, emerge under different lodging pressures. In addition, the predicted lodging score based on the QTL haplotype vs. the phenotypic lodging score suggests that different QTL could be relevant at low- compared to high-lodging scores ranges. Encouragingly, despite the genetic complexity of lodging, our data showed that lines with a larger number of more favourable alleles lodged less than lines with fewer favourable alleles in all populations and environments, suggesting that these markers will be useful in selecting for low-lodging germplasm.

### Conclusions

Lodging is a complex, quantitatively regulated trait underpinned by different plant characteristics and influenced by the environment in many crops. Our 5-year wheat pre-breeding journey led from donor identification to low-lodging high-yielding germplasm in adapted backgrounds and DNA markers, ready for industry uptake, through the development of multi-donor × elite-based crosses. Further value in the approach utilised in the current study lies in the ability to provide a usable set of markers representing different lodging pressure, and future work could focus on a few regions to look for new alleles across a range of donor sources. The combination of donor selection for highly heritable underpinning traits, refined DNA markers, environment manipulation and high-throughput evaluation of lodging score could be a route to support increasing attainable yield in breeding programs of different crops. The fact that the combination of traits responsible for standability and underpinning QTL differs under contrasting lodging pressure needs to be acknowledged to decide on the best route for rapid breeding progress.

## Supplementary Information

Below is the link to the electronic supplementary material.

Supplementary file1 (XLSX 2075 KB)

## Data Availability

Data would be made available on request upon approval by the GRDC and CSIRO who cofounded the research.
